# Pathogenesis and management of *TRPV3*-related Olmsted syndrome

**DOI:** 10.3389/fgene.2024.1459109

**Published:** 2024-12-11

**Authors:** Antong Lu, Kezhen Li, Cong Huang, Bo Yu, Weilong Zhong

**Affiliations:** ^1^ Department of Dermatology, Peking University Shenzhen Hospital, Shenzhen, China; ^2^ Shenzhen Key Laboratory for Translational Medicine of Dermatology, Biomedical Research Institute, Shenzhen Peking University-The Hong Kong University of Science and Technology Medical Center, Shenzhen, China; ^3^ Institute of Dermatology, Shenzhen Peking University-The Hong Kong University of Science and Technology Medical Center, Shenzhen, China; ^4^ Shantou University Medical College, Shantou, China; ^5^ Shenzhen University Medical School, Shenzhen, China

**Keywords:** Olmsted syndrome, TRPV3, hyperkeratosis, itch, mutation

## Abstract

Olmsted syndrome is characterized by symmetrically distributed, destructive, inflammatory palmoplantar keratoderma with periorificial keratotic plaques, most commonly due to gain-of-function mutations in the transient receptor potential vanilloid 3 (TRPV3) gene, which involves multiple pathological functions of the skin, such as hyperkeratosis, dermatitis, hair loss, itching, and pain. Recent studies suggest that mutations of *TRPV3* located in different structural domains lead to cases of varying severity, suggesting a potential genotype-phenotype correlation resulting from TRPV3 gene mutations. This paper reviews the genetics and pathogenesis of Olmsted syndrome, as well as the potential management and treatment. This review will lay a foundation for further developing the individualized treatment for TRPV3-related Olmsted syndrome.

## Introduction

Olmsted syndrome (OS) is a rare hereditary dermatosis characterized by symmetrically distributed, destructive, inflammatory palmoplantar keratoderma (PPK) with periorificial keratotic plaques. The PPK could be very mild, focal, non-mutilating to extremely severe, diffuse and mutilating (e.g., spontaneous amputation). Periorificial hyperkeratosis could be noted in perioral, perinasal, perianal areas, perineum, and ear meatus. Other documented features include hair and/or nail abnormality. It is also accompanied by varying degrees of pruritus, pain, and even erythermalgia (Duchatelet et al., 2014; Duchatelet and Hovnanian, 2015). While the actual number of cases may be underreported, the prevalence is estimated to be below 1 in 1,000,000. This disease affects both genders with male predisposition, which accounts for approximately 63% of reported OS patients (Duchatelet et al., 2014).

Histopathological analysis of skin samples reveals epidermal hyperplasia, orthohyperkeratosis, focal parakeratosis, hypergranulosis, acanthosis, and inflammatory infiltration in the upper dermis (Duchatelet and Hovnanian, 2015). These findings are non-specific and provide limited guidance for diagnosis. In the absence of specific biological markers, molecular genetics is the most effective method for establishing a diagnosis, particularly when the clinical presentation is atypical (Duchatelet and Hovnanian, 2015).

The causative genes of OS include membrane-bound transcription factor peptidase, site 2 (MBTPS2) gene or p53 effector related to PMP-22 (PERP), and transient receptor potential vanilloid 3 (TRPV3). MBTPS2 encodes site-2 protease (S2P) that is an integral membrane protein crucial for regulating membrane-bound transcription factors (Caengprasath et al., 2021), while PERP is responsible for coding a vital component of desmosomes (Duchatelet et al., 2019). Although *MBTPS2* for X-linked recessive OS and *PERP* for autosomal dominant OS have been reported, the most common etiology of OS is gain-of-function mutations in *TRPV3* with different patterns of inheritance.

However, the mechanism by which TRPV3 causes OS remains unclear, and there needs to be more comprehensive summaries regarding the progress of potential therapeutic drugs. Previous reviews have elucidated the clinical manifestations and differential diagnosis of OS. This review summarizes the pathogenic mechanisms and therapeutic advancements related to OS associated with *TRPV3* mutations.

## Methods

This review is based on literature search using PubMed. To identify all relevant literature related to OS and summarize reported cases, a computerized search of the PubMed database was performed, including all studies published before 1 November 2023, using the search terms “TRPV3” and “Olmsted syndrome.”

## Characteristics of TRPV3 channel

The TRPV3 channel is a symmetrical homotetramer, with each subunit consisting of an intracellular amino-terminal, carboxyl-terminal, linker region, and six transmembrane helices (S1-S6). The carboxyl-terminal contains a highly conserved transient receptor potential (TRP) domain (Zhong et al., 2021; Singh et al., 2018).

TRPV3 is expressed in various human tissues, including the skin, brain, spinal cord, dorsal root ganglia (DRG), and testes (Xu et al., 2002). In the skin, TRPV3 is highly expressed in the keratinocytes of the stratum basale and outer root sheath (ORS) of hair follicles (Peier et al., 2002; Park et al., 2017; Moqrich et al., 2005; Borbiro et al., 2011; Asakawa et al., 2006). The TRPV3 channel is activated at innocuous temperatures, ranging from 31°C–39°C, and it remains active within a noxious temperature range (Liu et al., 2011). Chemical activators for the TRPV3 channel include spice extracts (such as camphor, carvacrol, thymol, and eugenol), synthetic agents (including 2-aminoethoxydiphenyl borate, i.e., 2-APB), and endogenous ligand farnesyl pyrophosphate (FFP) (Xu et al., 2002; Peier et al., 2002; Xu et al., 2006; Vogt-Eisele et al., 2007; Smith et al., 2002; Luo and Hu, 2014; Bang et al., 2010). Repeated exposure to heat or chemical stimuli leads to receptor sensitization through gating hysteresis (Moqrich et al., 2005; Liu et al., 2011; Xiao et al., 2008), and the combined application of various stimuli acts synergistically (Moqrich et al., 2005; Grubisha et al., 2014). Conversely, factors like adenosine triphosphate (ATP), Mg^2+^, and phosphatidylinositol 4,5-bisphosphate (PIP2) can inhibit TRPV3 sensitivity (Kim et al., 2013; Grandl et al., 2010; Chung et al., 2005). Studies in mouse and human keratinocyte cell lines show that TRPV3 activation induces whole-cell currents, intracellular calcium increases, or nitric oxide production, and release of inflammatory mediators like interleukin-1α (IL-1α) and prostaglandin E2 (PGE2) (Moqrich et al., 2005; Xu et al., 2006; Yuspa et al., 1983; Miyamoto et al., 2011; Luo et al., 2012; Huang et al., 2008; Hu et al., 2006; Chung et al., 2004a; Chung et al., 2004b; Cheng et al., 2010; Cao et al., 2012).

Abundant evidence suggests that TRPV3 is involved in the natural development of hair. In mice, knockout of the TRPV3 gene results in irregular hair (bent and curled) and follicular keratosis, indicating that TRPV3 is responsible for maintaining normal hair morphology (Moqrich et al., 2005; Song et al., 2021). Numerous studies have shown that TRPV3 can significantly promote cell proliferation. Aijima et al. (2015) found that TRPV3 activation induces oral epithelial proliferation, promoting wound healing. Further researches revealed that TRPV3 greatly enhances keratinocyte proliferation through an epidermal growth factor receptor (EGFR)-dependent signaling pathway (Wang et al., 2021; He et al., 2015). Additionally, it has been found that TRPV3 is involved in maintaining skin barrier function and vascular formation (Cheng et al., 2010).

## Aetiology

### Genetics

**FIGURE 1:**
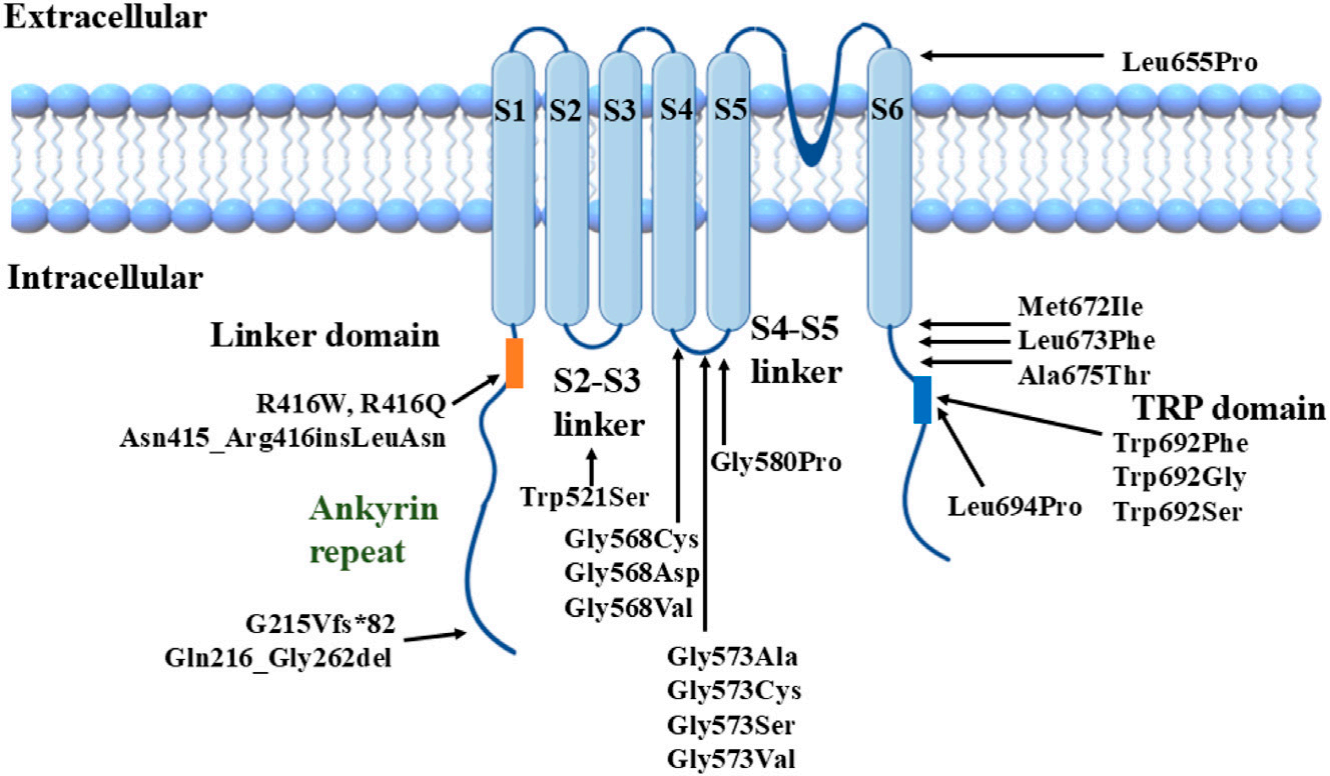
Structural model of TRPV3 channel with reported mutations.

The total number of OS cases is 100 (Duchatelet and Hovnanian, 2015; Zhong et al., 2021; Lu et al., 2021; Chiu et al., 2020). This paper mainly focuses on *TRPV3* mutation-related OS, over 40 cases of OS with *TRPV3* mutations are summarized in Supplementary Table S1. Though most reported cases are sporadic, familial cases with different genetic patterns (autosomal dominant, autosomal recessive, or X-linked inheritance) have also been documented. At least 22 *TRPV3* mutations have been identified in OS patients (Zhong et al., 2021) (Figure 1). These mutations are located in different structural domains and can lead to OS cases of varying severity, suggesting a potential genotype-phenotype correlation resulting from *TRPV3* gene mutations (Chiu et al., 2020; Wilson et al., 2015; Ni et al., 2016; Noakes, 2021). For instance, patients carrying p.Gly573Cys or p.Trp692Gly heterozygous mutations exhibit more severe hyperkeratosis compared to OS patients carrying p.Gln580Pro or p.Met672Ile heterozygous mutations. *In vitro* studies have shown that all four mutations mentioned above can reduce the activity of HaCaT cells and induce apoptosis, with significantly enhanced cytotoxicity observed for p.Gly573Cys and p.Trp692Gly compared to p.Gln580Pro and p.Met672Ile (Ni et al., 2016).

We previously found a correlation between TRPV3 genotype and OS phenotype, i.e., mutations located in the S4-S5 linker region and the TRP domain of TRPV3 significantly enhance channel function and lead to severe phenotypes. In contrast, the other mutations have a milder effect on channel function and disease phenotype (Zhong et al., 2021). We selected three TRPV3 variants, p.Arg416Gln, p.Leu673Phe, and p.Gln692Ser, representing mild, moderate, and severe clinical phenotypes of OS (especially the degree of keratinization), respectively, for functional studies. *In vitro* cytotoxicity experiments showed that mutant p.Trp692Ser was the most cytotoxic, p.Arg416Gln was the weakest, and p.Leu673Phe was intermediate. *In vitro* electrophysiological experiments also revealed that the increase in voltage sensitivity and the elevation of channel basal opening probability were most significant in p.Trp692Ser and not in p.Arg416Gln, whereas the electrophysiological properties of p.Leu673Phe were intermediate between p.Trp692Ser and p.Arg416Gln.

### Pathogenesis

#### Hyperkeratosis

Even before the identification of the causative genes for OS, multiple studies (Raskin and Tu, 1997; Nofal et al., 2010; Kress et al., 1996) reported that in keratinocytes of OS, there is inadequate expression of mature epidermal proteins, persistent presence of basal layer keratins, and an increased expression of the proliferation marker Ki-67 in the basal layer and suprabasal layers (Tao et al., 2008; Requena et al., 2001; Elise Tonoli et al., 2012). Consequently, the involved region remains immature and continues to proliferate inappropriately. This excessive epithelial proliferation can lead to hyperkeratosis. In 2012, Lin et al. (2012) found that gain-of-function mutations in TRPV3 (p.Gly573Cys, p.Gly573Ser, and p.Trp692Gly) lead to sustained channel opening, continuous influx of Ca^2+^ into cells, causing calcium overload and inducing increased apoptosis of keratinocytes, which may result in the hyperkeratosis observed in OS. In 2015, transcriptomic and proteomic studies on skin lesions and non-lesions of OS patients carrying the p.Gln580Pro mutation revealed downregulation of proteins involved in keratinocyte differentiation and cell-cell connections, while proteins related to keratinocyte proliferation and cell death were upregulated, suggesting that TRPV3 mutations may disrupt the balance between keratinocyte proliferation and differentiation, impairing cell adhesion function (He et al., 2015).

In 2017, further *in vitro* studies found that TRPV3 mutants (p.Gly573Ala, p.Gly573Cys, p.Gly573Ser, and p.Trp692Gly) exhibited reduced membrane surface localization in HaCaT cells, being mainly localized to the endoplasmic reticulum. These mutations also reduced cell adhesion, abnormal lysosomal and endoplasmic reticulum functions, and mitochondrial aggregation (Yadav and Goswami, 2017). Further research found that in cells expressing the p.Gly568Cys and p.Gly568Asp mutants, there was a significant reduction in the number of lysosomes (Jain et al., 2022). The p.Gly568Cys mutant showed decreased lysosomal movement, while the p.Gly568Asp mutant cells exhibited reduced lysosomal acidification levels. Notably, the Ca^2+^ levels within lysosomes closely relate to their pH regulation (Scott and Gruenberg, 2011; Cao et al., 2017). This is because lysosomes need to retain more Ca^2+^ to maintain their low pH, and uncontrolled release of Ca^2+^ can lead to difficulties in lysosomal lumen acidification (Koh et al., 2019). Therefore, the p.Gly568Cys and p.Gly568Asp mutants may act as Ca^2+^ leak channels in lysosomes to varying degrees (Jain et al., 2022). However, whether similar phenomenon could be found in other subcellular organelles with other mutations in different TRPV3 structural domains needs to be further investigated. Meantime, it may also disrupt the balance between keratinocyte proliferation and differentiation, thereby contributing to the development of OS.

Increasing evidence suggests that the wild-type TRPV3 can regulate hair development and epidermal keratinization by activating the EGFR pathway in keratinocytes, and different degrees of activation of the wild-type TRPV3 channel may have various regulatory effects on keratinocytes. In 2010, researchers found that mice with keratinocytes-specific knockout of the *Trpv3* gene had wavy hair and defects in the skin barrier (Cheng et al., 2010). Further studies revealed that TRPV3 forms a signaling complex with transforming growth factor-α (TGF-α)/EGFR. The constitutive low-level activity of TRPV3 can activate EGFR at a low level, and downstream activation of ERK can further sensitize TRPV3, forming a positive feedback loop. This loop plays a crucial role in normal hair development, terminal differentiation of suprabasal keratinocytes, and the formation of the skin barrier cornified envelope (Cheng et al., 2010). Recent research reported that lower concentrations (30 μM or 100 μM) of carvacrol can moderately activate TRPV3, which then promotes proliferation in HaCaT cells and epidermal hyperplasia in mice through the activation of the EGFR/PI3K/NF-κB pathway (Grubisha et al., 2014).

Several studies have found that higher concentrations of TRPV3 agonists (such as 300 μM carvacrol) can lead to excessive activation of the wild-type TRPV3 channel, inducing apoptosis/death of keratinocytes (Zhang et al., 2019; Yan et al., 2019; Szollosi et al., 2018). Blocking NF-κB can further increase cell death (Szollosi et al., 2018). Therefore, some researchers propose that the excessive activation of the TRPV3 channel may also promote keratinocytes proliferation through the EGFR/PI3K/NF-κB pathway. However, this effect is masked by the massive cell death (apoptotic effect) caused by excessive channel activation (Grubisha et al., 2014).

#### Dermatitis

Previous studies on mouse models also suggest that TRPV3 mediates the release of pro-inflammatory cytokines, including IL-1α (Xu et al., 2006) and PGE2 (Huang et al., 2008), essential mediators of skin inflammation. Lin et al. (2012) found that the mutated human TRPV3 channel exhibited constitutive activity when expressed in HEK293 cells and led to the death of transfected HEK293 cells, as well as apoptosis of keratinocytes in skin biopsy sections from affected individuals. Studies indicate that the *in vitro* activation of TRPV3 and gain-of-function mutations can lead to significant skin inflammation in humans and rodents (Song et al., 2021; Lin et al., 2012; Szollosi et al., 2018; Yoshioka et al., 2009; Danso-Abeam et al., 2013). For example, recent findings show that the excessive activation of wild-type TRPV3 channels induced by high concentrations (300 μM) of carvacrol induces KC apoptosis and activates the NF-κB pathway to promote the release of various inflammatory mediators (Szollosi et al., 2018). Pathological examination of skin lesions in OS patients reveals psoriasis-like epidermal hyperplasia and infiltration of inflammatory cells in the upper dermis (Lin et al., 2012). Skin thickening, inflammatory reactions, and itching can be observed in WBN/Kob-Ht rats carrying the p.Gly573Cys mutation and DS-*Nh* mice carrying the p.Gly573Ser mutation (Yoshioka et al., 2009). As most OS patients have heterozygous mutations in TRPV3, to better simulate the clinical situation, *Trpv3^+/p.Gly568Val^* mice were created. Clinical manifestations include skin thickening and reduced hair, and histopathology shows significant epidermal hyperplasia and pronounced infiltration of inflammatory cells (Song et al., 2021).

#### Hair loss

In cultured human ORS keratinocytes, activation of TRPV3 induces membrane currents, increases intracellular calcium ions, inhibits cell proliferation, induces cell apoptosis, suppresses hair shaft elongation, and promotes premature regression of hair follicles. These cellular effects, including hair growth inhibition, are blocked by siRNA-mediated knockdown of TRPV3 (Borbiro et al., 2011). Gain-of-function mutations in TRPV3 alter the hair growth cycle in mice, namely, the process of follicular growth (anagen phase) and regression (telogen phase). In DS-*Nh* mice, genes related to the hair growth cycle, such as keratin-associated proteins 16–1, 16–3, and 16–9, are downregulated in the skin, and the anagen phase persists (Imura et al., 2007).

Song et al. (2021) generated an OS mouse model by introducing the p.Gly568Val point mutation in the corresponding TRPV3 locus in mice. They found that these mice exhibited alopecia, which was associated with premature differentiation of hair follicle keratinocytes, characterized by early senescence of hair keratin and trichohyalin, increased production of deiminated proteins, enhanced apoptosis, and reduced expression of transcription factors known to regulate hair follicle differentiation (*Foxn1, Msx2, Dlx3, and Gata3*). These abnormalities occurred in the inner root sheath and proximal hair shaft regions, where TRPV3 is highly expressed and associated with impaired hair shaft and canal formation. Mutant *Trpv3* mice showed increased proliferation of ORS, accelerated hair cycle, reduced hair follicle stem cells, and miniaturization of regenerative hair follicles.

#### Itch

The first signs of itch mediated by TRPV3 come from mutant mice. DS-*Nh* mice and WBN/Kob-Ht rats, both carrying autosomal dominant TRPV3 mutations, exhibit symptoms of atopic dermatitis (AD), including itchiness (Watanabe et al., 2003; Asakawa et al., 2005). Yoshioka et al. (2009) developed transgenic mice expressing mutant p.Gly573Ser in TRPV3 and found that these mice spontaneously exhibited scratching behavior after developing dermatitis, indicating that TRPV3 gene mutations cause itching. Yang et al. (2015) applied a TRPV3 agonist, carvacrol, to human burn scars and effectively induced itchiness. Enhanced TRPV3 expression was also detected in the epidermis of burn scars accompanied by itchiness (Kim et al., 2020). Compared to AD patients without itchiness, AD patients with itchiness showed increased levels of TRPV3 mRNA transcripts (Yamamoto-Kasai et al., 2012). These findings strongly support the hypothesis that TRPV3 plays a crucial role in chronic itch associated with skin dryness in mice and human.

Upon heat stimulation or activation by TRPV3 agonists, activated TRPV3 induces itchiness by promoting the secretion of thymic stromal lymphopoietin (TSLP), nerve growth factor (NGF), PGE2, and IL-33 by keratinocytes (Seo et al., 2020). Inflammation may also play an essential role in the propagation of TRPV3-mediated itchiness, as inflammatory factors such as IL-31 induce high expression of brain natriuretic peptide (BNP), activate TRPV3, upregulate expression of Serpin E1, and cause skin itching (Asakawa et al., 2006; Meng et al., 2018; Larkin et al., 2021).

Additionally, the lack of TRPV3 in keratinocytes impaired the function of protease-activated receptor 2 (PAR2), leading to reduced activation of neurons by PAR2 agonists and decreased scratching behavior. Zhao et al. (2020) found that TRPV3 and PAR2 were upregulated in skin biopsy tissues from patients and mice with AD, and inhibition of TRPV3 and PAR2 reduced scratching and inflammatory responses in a mouse model of AD.

#### Pain

Over the past decade, extensive research has been conducted on the role of TRPV3 in pain sensation. However, few studies have provided conclusive evidence for TRPV3-mediated pain. TRPV3 is expressed in nociceptive neurons. Therefore, overactivation of TRPV3 may also lead to excessive production of local inflammatory cytokines, affecting the somatosensory system and resulting in peripheral neuropathic pain. Additionally, ATP released by TRPV3-mediated keratinocytes can activate purinergic receptors in dorsal root ganglion neurons, affecting nociceptive function. Consistent with this, transgenic mice overexpressing TRPV3 in skin keratinocytes showed increased pain sensitivity (Huang et al., 2008). As a thermosensitive ion channel, TRPV3 is suspected to be associated with thermal nociception, as demonstrated by the disrupted response of *Trpv3* knockout mice to acute noxious heat (>48°C) in water immersion and hot plate (>52°C) experiments (Moqrich et al., 2005). Apart from thermal nociception, TRPV3 also mediates chemically induced acute nociceptive responses. FPP, an intermediate metabolite of the mevalonate pathway, is a selective TRPV3 agonist. Injection of FPP into the paw of carrageenan-pretreated mice induces rapid pain-related behaviors but not in non-sensitized mice. Knockdown of TRPV3 in the paws of mice using TRPV3-shRNA significantly alleviates FPP-induced pain-related behaviors but does not affect pain induced by the TRPV1 activator capsaicin (Bang et al., 2010). However, the role of TRPV3 in thermal nociception remains controversial. Huang et al. (2011) demonstrated that TRPV3-deficient mice showed no changes in acute thermal pain or inflammatory thermal hyperalgesia, suggesting that TRPV3 may not be a significant factor in thermal nociception.

## Management including treatments

### Traditional management and treatments

Various treatment methods have been attempted in the past to reduce excessive keratinization (Supplementary Table S2). Topical therapies include emollients (white petrolatum), keratolytic agents (urea, salicylic acid), wet dressings, boric acid, tar, retinoids, shale oil, corticosteroids, and calcineurin inhibitors, but their efficacy is generally poor to moderate. In some OS patients, systemic use of retinoids (acitretin, isotretinoin), corticosteroids, or methotrexate has resulted in varying degrees of relief from poor to moderate (Ueda et al., 1993; Tang et al., 2012; Rivers et al., 1985; Hausser et al., 1993). A few patients have also undergone repeated partial or full-thickness excision with skin grafting of the palmar-plantar keratoderma, but keratinization often recurs after initial improvement (Bedard et al., 2008). Regarding specific treatments for pain and itching, analgesics and topical lidocaine may be effective. Still, for patients with severe pain, especially when erythromelalgia or neuropathic pain is present, anti-inflammatory drugs, anticonvulsants (carbamazepine, gabapentin, pregabalin), tricyclic antidepressants (amitriptyline, desipramine), and ultimately opioid analgesics (tramadol) are needed to alleviate their pain (Duchatelet and Hovnanian, 2015). Soaking the affected areas in cold water for a prolonged period can relieve pain. However, these treatments are ineffective in preventing disease progression.

### EGFR inhibitors and mTOR inhibitors

**FIGURE 2:**
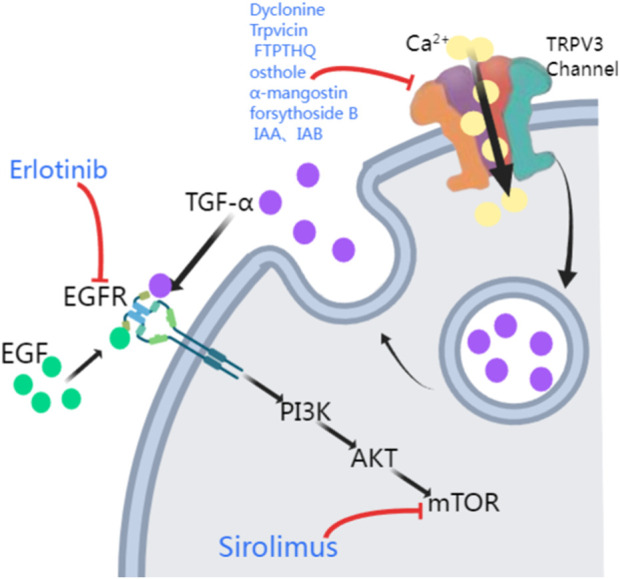
Potential treatments of *TRPV3* mutation-related OS. The activation of TRPV3 channels leads to Ca^2+^ influx, which causes a surge in intracellular Ca^2+^ signaling, promoting the release of TGF-α. TGF-α and EGF then activate their receptor EGFR, activating the EGFR/PI3K/AKT/mTOR cascade. TRPV3 antagonists (Dyclonine, Trpvicin, FTP-THQ, osthole, α-mangostin, forsythoside B, IAA, and IAB) inhibit the TRPV3 channel. Erlotinib and Sirolimus inhibit EGFR and mTOR respectively (PI3K: phosphatidylinositol-3-kinase; AKT: protein kinase B, PKB).

The TRPV3 gene encodes a channel that allows calcium ion influx into keratinocytes. As mentioned previously, downstream of EGFR signaling pathway plays an important role in maintaining epidermal homeostasis by forming a complex with TRPV3 (Zhong et al., 2021; Cheng et al., 2010). In 2020, Greco et al. (2020) demonstrated relief from *TRPV3* mutation-associated PPK, pain, and hyperkeratosis in three OS patients treated with the EGFR inhibitor erlotinib. These patients showed complete relief from hyperkeratosis and pain within 3 months of erlotinib therapy, leading to better growth and social engagement for the benefits lasted for 12 months (Greco et al., 2020). Amplification of EGFR leads to the activation of many downstream kinases, including mammalian targets of rapamycin (mTOR) (Wee and Wang, 2017) (Figure 2). Zhang et al. (2020) proposed that *TRPV3* mutations are associated with the pathogenesis of OS, possibly involving the EGFR-mTOR cascade, leading to skin inflammation and hyperproliferation. Oral administration of the mTOR inhibitor sirolimus in children with OS resulted in significant improvement in skin inflammation and periorificial hyperkeratotic plaques, while a substantial reduction in PPK and associated functional limitations was observed with the clinical response to erlotinib. In this study, two patients were first treated with sirolimus, which resulted in considerable improvement in erythema and periorificial hyperkeratosis. Following the transition to erlotinib, significant progress in PPK was also noted. The other two OS patients, treated only with erlotinib, had rapid PPK resolution and better quality-of-life scores (Bedard et al., 2008). In forementioned two studies, all patients presented sustained improvements in itching and pain, without severe side effects reported. EGFR inhibitors (e.g., erlotinib) and mTOR inhibitors (e.g., sirolimus) may be potential treatment options for OS hyperkeratosis, showing clinical improvements in hyperkeratosis, pain, and other symptoms in OS patients, which suggests that the EGFR and mTOR pathways are key therapeutic targets (Zhong et al., 2021; Greco et al., 2020; Zhang et al., 2020; Kenner-Bell et al., 2010; Fogel et al., 2015; Buerger et al., 2017). However, more case series and further basic researches are needed to elucidate the importance of EGFR and mTOR in the pathogenesis of *TRPV3*-related OS and the subsequent therapeutic effect of respective signaling inhibitors, thus contributing to the individualized therapy respective to different *TRPV3* mutations.

### TRPV3 antagonists

Gain-of-function mutations in the TRPV3 gene are responsible for increased apoptosis of affected individuals’ keratinocytes and excessive skin keratinization. Therefore, TRPV3 antagonists may be a practical therapeutic approach for treating OS patients carrying such mutations. Liu et al. (2021) reported that the clinical drug dyclonine inhibits TRPV3, relieving skin inflammation. However, due to the relatively low affinity of dyclonine for TRPV3, the information provided by the structure-based drug optimization is limited. Fan et al. (2023) found that the TRPV3 antagonist Trpvicin effectively relieved OS-like symptoms in a mouse model. However, particularly in chronic itching and hair loss mouse models, it is necessary to conduct pharmacokinetic studies on mouse skin to detect drug exposure further to ensure that the observed efficacy is due to TRPV3 blockade by Trpvicin. Hydra Inc.’s FTP-THQ is an efficient and selective *in vitro* antagonist of recombinant and native TRPV3 receptors, with almost no activity against TRPV1, TRPV4, TRPM8, and TRPA1 (Grubisha et al., 2014). FTP-THQ has now been evaluated *in vitro* for its effects on m308 keratinocytes, confirming its ability to block ATP and GM-CSF release by aggregating DPBA and 2-APB, known to selectively activate TRPV3 in these cells (Luo and Hu, 2014).

Many herbal extracts also inhibit TRPV3, including osthole, α-mangostin, forsythoside B, and isomers of isochlorogenic acid (Figure 2). Research has shown that osthole, an active ingredient in fructus cnidii, has been used in traditional Chinese medicine to treat eczema. Osthole inhibits TRPV3, and Neuberger et al. (2021) revealed two osthole binding sites on the transmembrane region of TRPV3, which overlap with the binding site of the agonist 2-APB. So far, osthole and its derivatives have been limited in disease treatment due to the need for high concentrations and their action on multiple targets (Kim et al., 2020). α-Mangostin, an extract from the pericarp of *Garcinia mangostana*, effectively inhibits TRPV3, reducing calcium influx and cytokine release, protecting cells from TRPV3-induced death, and has inhibitory effects on both wild-type and mutant TRPV3 (Dang et al., 2023). Forsythoside B inhibits channel currents activated by the agonist 2-aminoethyl diphenyl borate, and its pharmacological inhibition of TRPV3 significantly alleviates acute itching induced by carvacrol or histamine, as well as chronic itching caused by acetone-ether-water in mice with dry skin. Additionally, forsythoside B can prevent cell death in human keratinocytes expressing the TRPV3 p.Gly573Ser gain-of-function mutation or in HEK293 cells or naturally immortalized non-carcinogenic keratinocytes in the presence of the TRPV3 agonist carvacrol (Zhang et al., 2019). Forsythoside B also significantly reverses the inhibitory effect of carvacrol on hair growth (Yan et al., 2019). Two natural isomers of isochlorogenic acid, IAA and IAB, act as TRPV3 channel gating modifiers, selectively inhibiting channel currents by reducing channel opening probability. IAA and IAB can alleviate excessive activation of TRPV3-induced keratinocyte death, ear swelling, and chronic itching (Qi et al., 2022).

## Conclusion and future perspectives

This review summarizes the pathogenic mechanisms and therapeutic advances of OS associated with *TRPV3* mutations. Despite obtaining compelling evidence from TRPV3 mutant alleles and transgenic mice, the low selectivity of TRPV3 antagonists has diminished reliability. Therefore, future research is required to develop TRPV3 antagonists with higher selectivity. In conclusion, this review elucidates the main clinical symptoms and mechanisms of *TRPV3* mutation-related OS, laying the foundation for the individual treatment of OS.

## Glossary

AD: Atopic dermatitis
AKT (PKB): Protein kinase B
ATP: Adenosine triphosphate
BNP: Brain natriuretic peptide
DPBA: Dipeptide boronic acid
DRG: Dorsal root ganglia
EGFR: Epidermal growth factor receptor
FPP: Farnesyl pyrophosphate
GM-CSF: Granulocyte-macrophage colony stimulating factor
IA: Isochlorogenic acid
KC: Keratinocyte
MBTPS2: Membrane-bound transcription factor peptidase, site-2
mTOR: Mammalian targets of rapamycin
NGF: Nerve growth factor
NF-κB: Nuclear factor kappa-B
ORS: Outer root sheath
OS: Olmsted syndrome
PAR2: Protease-activated receptor 2
PERP: p53 effector related to PMP-22
PGE2: Prostaglandin E2
PIP2: Phosphatidylinositol 4,5-bisphosphate
PI3K: Phosphatidylinositol-3-kinase
PPK: Palmoplantar keratoderma
S2P: Site-2 protease
TGF-α: Transforming growth factor-α
TRP: Transient receptor potential
TRPA1: Transient receptor potential ankyrin 1
TRPM8: Transient receptor potential melastatin 8
TRPV3: Transient receptor potential vanilloid 3
TSLP: Thymic stromal lymphopoietin
2-APB: 2-aminoethoxydiphenyl borate
