# Supplementary material for: Pathogenesis and management of TRPV3-related Olmsted syndrome
Source: Front Genet. 2024 Dec 11;15:1459109. doi: 10.3389/fgene.2024.1459109 (PMC11694452; doi:10.3389/fgene.2024.1459109)
Supplement: Supplementary file 1 [file Table1.docx]

**Supplementary Material**

**Table S1.** Characteristics of previously documented Olmsted Syndrome patients with *TRPV3* mutations

| Patient | Sex | Palmoplantar keratoderma (PPK) and periorificial keratoderma | Hair abnormalities | Lesional symptoms | Mutation | | |
| --- | --- | --- | --- | --- | --- | --- | --- |
|  |  |  |  |  | Nucleotide  (Amino acid) | Exon | Inheritance of mutation |
| Lin *et al.,* 2012 | F | Moderate PPK with constricting digit bands; severe periorificial keratoderma | Severe alopecia | Moderate itch, and pain | c.1717G>A  (p.Gly573Ser) | 13 | *De novo* |
|  | F | Mild PPK and periorificial keratoderma; | Moderate alopecia | Moderate itch, and pain | c.1717G>T  (p.Gly573Cys) | 13 | *De novo* |
|  | F | Severe PPK with spontaneous digit amputation; severe periorificial keratoderma | Severe alopecia | Moderate itch, and pain | c.1717G>A  (p.Gly573Ser) | 13 | Autosomal-dominant |
|  | F | Severe PPK with constricting digit bands and spontaneous digit amputation; moderate periorificial keratoderma | Moderate alopecia | Moderate itch, and pain | c.1717G>A  (p.Gly573Ser) | 13 | *De novo* |
|  | M | Moderate PPK with constricting digit bands; moderate periorificial keratoderma | Mild alopecia | Moderate itch, and pain | c.2074T>G  (p. rp692Gly) | 15 | *De novo* |
|  | F | Mild PPK with constricting digit bands; mild periorificial keratoderma | Dry curly hair | Moderate itch, and pain | c.1717G>A  (p.Gly573Ser) | 13 | *De novo* |
| Lai-Cheong *et al.,* 2012 | M | Severe mutilating PPK with flexion deformities of the fingers; mild periorificial keratoderma with natal cleft | Dry and lusterless hair | Pain, functional impairment | c.1717G>A  (p.Gly573Ser) | 13 | *De novo* |
| Danso-Abeam *et al.,* 2013 | M | Mild and diffuse PPK; severe periorificial keratoderma | Severe alopecia | Severe itch and pain | c.1718G>C  (p.Gly573Ala) | 13 | *De novo* |
| Kariminejad *et al.,* 2014 | M | Severe and diffuse PPK with flexion contractures and autoamputation; moderate periorificial keratoderma | Moderate hair abnormalities with a lack of body hair | Severe itch and pain | c.2076G>C  (p.Trp692Cys) | 15 | *De novo* |
| Patient | Sex | Palmoplantar keratoderma (PPK) and periorificial keratoderma | Hair abnormalities | Lesional symptoms | Mutation | | |
|  |  |  |  |  | Nucleotide (Amino acid) | Exon | Inheritance of mutation |
| Eytan *et al.,* 2014 | F | Severe and diffuse PPK with fingers deformities; severe periorificial keratoderma | Sparse hair | Itch and pain | c.1562G>C  (p.Trp521Ser) | 12 | Autosomal recessive |
| Duchatelet *et al.,* 2014a | M | Severe and diffuse PPK | Thin and dry hair | Severe itch and pain (erythermalgia) | c.1702G>T  (p.Gly568Cys); c.784+1G>A (p. Gln216_Gly262del) | 13 | Autosomal recessive |
|  | M | Focal and moderate PPK | Thin and dry hair | Severe itch and moderate pain (erythermalgia) |  | 13 | Autosomal recessive |
| Duchatelet *et al.,* 2014b | F | Severe and diffuse PPK | Fine, dry, curly and unmanageable hair | Severe itch and pain (erythermalgia) | c.2017C>T  (p.Leu673Phe) | 15 | *De novo* |
| Wilson *et al.,* 2015 | F | Severe and focal PPK | Fine hair | Severe pain | c.1717G>T  (p.Gly573Cys) | 13 | *De novo* |
|  | F | Moderate and focal PPK | Normal hair | Severe pain | c.1703G>T  (p.Gly568Val) | 13 | *De novo* |
|  | F | Moderate and focal PPK | Fine hair | Severe itch and pain | c.1703G>A  (p.Gly568Asp) | 13 | Autosomal-dominant |
|  | F | Moderate and focal PPK | Normal hair | Severe pain | c.1703G>A  (p.Gly568Asp) | 13 | Not reported |
|  | M | Moderate and focal PPK | Moderate, fragile, sparse hair | Severe pain (erythermalgia) | c.1717G>A  (p.Gly573Ser) | 13 | Autosomal-dominant |
|  | F | Moderate and focal PPK | Normal hair | Severe pain | c.1717G>A  (p.Gly573Ser) | 13 | *De novo* |
| He *et al.,* 2015 | M | Moderate and focal PPK | Normal hair | Not reported | c.1739A>C  (p.Gln580Pro) | 13 | Autosomal-dominant |
| Ni *et al.,* 2016 | M | Mild and focal PPK | Normal hair | Mild itch and pain (erythermalgia) | c.2016G>A  (p.Met672Ile) | 15 | Autosomal-dominant |
| Patient | Sex | Palmoplantar keratoderma (PPK) and periorificial keratoderma | Hair abnormalities | Lesional symptoms | Mutation | | |
|  |  |  |  |  | Nucleotide (Amino acid) | Exon | Inheritance of mutation |
| Agarwala *et al.,* 2016 | M | Severe, diffuse PPK with flexion deformities of the left wrist and fingers of both hands, painful pseudoainhum; moderate keratoderma around the mouth, ears and anus | Short hair | Severe pain | c.1246ins6 (p. Asn415_Arg416insLeuAsn) | 10 | *De novo* |
| Cao *et al.,* 2016 | M | Focal and mild PPK, mild flexion contraction of fingers | Not reported | Itch and pain (erythermalgia) | c.1702G>T (p.Gly568Cys); c.643+1G>T(p.Gly215Valfs*82) | 13 | Semi-dominant |
| Zhi *et al.,* 2016 | F | Severe, bilateral and mutilating PPK with flexion deformities, and constriction of digits; mild perioral keratoderma | Severe and diffuse alopecia | Not reported | c.1718G>T (p.Gly573Val) | 13 | Autosomal-dominant |
|  | M | Focal and mild PPK on the soles | Severe and diffuse alopecia | Not reported | c.1718G>T  (p.Gly573Val) | 13 | Autosomal-dominant |
| Takeichi *et al.,* 2017 | M | Focal and mild PPK on the big toes, the edges of the soles and right hand | Mild hypotrichosis with thin hair | Not reported | c.2017C>T  (p.Leu673Phe) | 15 | Autosomal-dominant |
|  | F | Severe and diffuse PPK with fingers showing moderate contracture |  | Severe itch |  | 15 | Autosomal-dominant |
| Nagai *et al.,* 2017 | M | Severe and diffuse PPK with flexion contractures of the fingers, a below knee amputation; mild periorificial keratoderma on the lower lip and sacral area | Moderate and localized alopecia | No itch but severe pain | c.1703G>T  (p.Gly568Val) | 13 | *De novo* |
| Choi *et al.,* 2018 | F | Severe bilateral PPK; moderate periorificial keratoderma around right ear | Moderate, thin and sparse hair | Severe itch and pain | c.1703G>T  (p.Gly568Val) | 13 | *De novo* |
| F. P.-C. Chiu *et al, 2020* | F | Severe bilateral mutilating PPK and pseudoainhum | Normal hair | Severe itch and pain | c.2023G>A  (p.Ala675Thr) | 15 | *De novo* |
| Patient | Sex | Palmoplantar keratoderma (PPK) and periorificial keratoderma | Hair abnormalities | Lesional symptoms | Mutation | | |
|  |  |  |  |  | Nucleotide (Amino acid) | Exon | Inheritance of mutation |
| Zhong *et al.*, 2021 | M | Moderate, asymmetric, diffuse and mutilating PPK in the left sole and hand; focal, nonmutilating PPK in the right side | Normal hair | None | c.1246C>T (p.Arg416Trp) | 10 | Autosomal-dominant |
| Zhong *et al.*, 2021 | M | Mild, asymmetric, focal and nonmutilating PPK in the right hand and foot; left side normal | Normal hair | Itch and moderate pain (erythermalgia) | c.1246C>T  (p.Arg416Trp) | 10 | Autosomal-dominant |
| Zhong *et al.*, 2021 | M | Mild, symmetric, focal and nonmutilating PPK; keratoderma in corners of mouth, coccygeal region | Lusterless and coarse hair | Itch and moderate pain | c.1247G>A  (p.Arg416Gln) | 10 | Autosomal-dominant |
| Zhong *et al.*, 2021 | M | Severe, symmetric, diffuse and mutilating PPK; moderate keratoderma in corners of mouth and right ear, perianal area | +; partial white hair | Severe itch and pain | c.1703G>T  (p.Gly568Val) | 13 | *De novo* |
| Zhong *et al.*, 2021 | M | Severe, symmetric, diffuse and mutilating PPK in planta; focal and nonmutilating PPK in palms; moderate keratoderma in coccygeal region | Normal hair | None | c.1703G>A  (p.Gly568Asp) | 13 | *De novo* |
| Zhong *et al.*, 2021 | F | Moderate, symmetric, diffuse and nonmutilating PPK with constriction digital bands | Normal hair | None | c.1964T>C  (p.Leu655Pro) | 15 | *De novo* |
| Zhong *et al.*, 2021 | M | Mild, symmetric, focal and nonmutilating PPK | Thin, coarse, and unmanageable hair with mild hypotrichosis | Mild itch and severe pain (erythermalgia) | c.2017C>T  (p.Leu673Phe) | 15 | *De novo* |
|  |  |  |  |  |  |  |  |
|  |  |  |  |  |  |  |  |
| Patient | Sex | Palmoplantar keratoderma (PPK) and periorificial keratoderma | Hair abnormalities | Lesional symptoms | Mutation | | |
|  |  |  |  |  | Nucleotide (Amino acid) | Exon | Inheritance of mutation |
| Zhong *et al.*, 2021 | M | Moderate, symmetric, diffuse and nonmutilating PPK with mild bone absorption of the terminal digits; mild keratoderma in perianal area | Moderate hypotrichosis, easily broken; eyebrows and eyelashes affected | Mild itch and pain | c.2075G>C  (p.Trp692Ser) | 15 | *De novo* |
| Zhong *et al.*, 2021 | M | Moderate, symmetric, and nonmutilating PPK in both feet; mild PPK in the terminal digits of both hands; keratoderma in corners of mouth | Normal hair | None | c.2081T>C  (p.Leu694Pro) | 15 | *De novo* |
| Lu J *et al.*, 2021 | F | Severe, symmetric, and mutilating PPK in both hands and feet | Normal hair | None | c.2016G>A  (p.Met672Ile) | 15 | *De novo* |
