# Supplementary material for: Pathogenesis and management of TRPV3-related Olmsted syndrome
Source: Front Genet. 2024 Dec 11;15:1459109. doi: 10.3389/fgene.2024.1459109 (PMC11694452; doi:10.3389/fgene.2024.1459109)
Supplement: Supplementary file 2 [file Table2.docx]

**Table S2.** Mechanisms of action of management and treatment of Olmsted syndrome

| Therapy | Name | Function and/or Mechanism | References |
| --- | --- | --- | --- |
| Topical therapies | White petrolatum | Skin moistens | Rivers JK *et al*., 1985  Hausser I *et al*., 1993  Ueda M *et al*., 1993  Tang L *et al*., 2012 |
|  | Urea  Salicylic acid  Wet dressings  Retinoids | Keratolysis |  |
|  | Boric acid  Tar | Antibacterial &  Anti-inflammatory |  |
|  | Corticosteroids | Anti-inflammatory |  |
|  | Tacrolimus | Immunosuppression | Duchatelet S. *et al*., 2015 |
|  | Topical lidocaine | Analgesia |  |
| Oral medicine | Tramadol | Analgesia | Duchatelet S. *et al*., 2015 |
|  | Carbamazepine  Gabapentin  Pregabalin | Anticonvulsion |  |
|  | Amitriptyline  Desipramine | Antidepression |  |
|  | Corticosteroids | Anti-inflammatory |  |
|  | Erlotinib | EGFR inhibitors | Greco C. *et al*., 2020 |
|  | Sirolimus | mTOR inhibitors | Fogel AL *et al*., 2015 |
| Surgery | Partial/full-thickness excision with skin grafting | | Bedard MS *et al*., 2008 |
| Potential drugs | Dyclonine  Trpvicin  FTP-THQ  Osthole  α-mangostin  Forsythoside B  IAA and IAB | TRPV3 antagonists | Grubisha O *et al*., 2014  Yan K *et al*., 2019  Fan J *et al*., 2023  Neuberger A *et al*., 2021  Dang TH *et al.*, 2023  Qi H *et al*., 2022 |
